# Multi-ancestry Genome-wide Association Study of Inpatient Opioid Dosing Following Knee or Hip Arthroplasty

**DOI:** 10.21203/rs.3.rs-7330342/v1

**Published:** 2025-08-21

**Authors:** Zeal Jinwala, Christal N. Davis, Jackson F. SooHoo, Million Veteran Program, Penn Medicine Biobank, Joel Gelernter, Rachel L. Kember, Rachel Vickers-Smith, Henry R. Kranzler

**Affiliations:** 1 Mental Illness Research, Education and Clinical Center, Crescenz Veterans Affairs Medical Center, Philadelphia, PA, USA; 2 Department of Psychiatry, University of Pennsylvania Perelman School of Medicine, Philadelphia, PA, USA; 3 Department of Epidemiology and Environmental Health, University of Kentucky College of Public Health, Lexington, KY, USA; 4 VA Connecticut Healthcare Center, West Haven, CT, USA; 5 Departments of Psychiatry, Genetics, and Neuroscience, Yale University School of Medicine, New Haven, CT, USA

## Abstract

Opioids are commonly prescribed to manage acute postoperative pain. However, individuals vary in their opioid dosing needs, with no validated biomarkers to guide prescribing. We used electronic health records (EHR) data from the Million Veteran Program sample to investigate individual differences in opioid analgesic dosing following knee (n=18,540) or hip (n=9,363) arthroplasty. We extracted data from pharmacy records on inpatient opioid medications to estimate average daily morphine milligram equivalent (MME) dosages and used genome-wide association studies (GWAS) to identify genetic variants associated with daily MME dosage, followed by a cross-ancestry and cross-phenotype meta-analysis. We identified two genome-wide significant loci in the African ancestry knee arthroplasty GWAS and three in the Admixed-American ancestry hip arthroplasty GWAS. Multiple other loci in African, Admixed-American, and European populations were nominally significant. The study provides a framework for the use of EHR data to examine the genetics of opioid dosing in post-operative care.

## Introduction

Opioid analgesics are commonly prescribed to manage acute postoperative pain. Although inter-individual variation in pain tolerance and opioid response contribute to decisions regarding which analgesic to use and at what dosage, there are no validated biomarkers to guide prescribing. This limits optimal dosing and analgesic efficacy.

In addition to its potential to improve clinical care, more precise opioid analgesic dosing could reduce the risk of opioid use disorder (OUD). The drive to manage pain aggressively between 1990 and 2010 produced a sharp increase in U.S. opioid prescriptions, one factor that fueled an epidemic of opioid misuse, dependence, and overdose deaths [39,43,8,45]. In the Veterans Health Administration (VHA), opioid prescribing nearly doubled over a decade [36] and was accompanied by a substantial rise in opioid-related overdose deaths [5]. Despite a recent widespread decline in opioid overdose deaths [1], the ongoing availability of illicit synthetic opioids, such as fentanyl, continues to pose a serious public health challenge [44]. Thus, there is a critical need for empirically-based, individualized opioid prescribing, particularly in acute care settings where individuals are often first exposed to opioids [32].

Despite well-recognized individual differences in opioid analgesic sensitivity, there is little known regarding the biological basis of this variability [24]. Candidate gene studies have yielded inconsistent findings [13,17,24,31,38]. Genome-wide association studies (GWAS) that have implicated genes such as *CREB1*, *TAOK3*, and *TRPC3*, were conducted in small samples (*n*s<650) and remain to be replicated [3,11,37]. Recent studies evaluating the utility of a machine learning algorithm to predict OUD based on candidate gene findings concluded that genetic variants in the algorithm are ineffective at predicting OUD [14,23]. A recent GWAS of the number of codeine prescriptions provided to patients identified 9 genome-wide significant (GWS) loci. There was also a significant correlation between genetic liability for OUD and the number of codeine prescriptions. However, the study was limited by its focus on a single opioid—codeine, lack of consideration of dosage, and the inclusion of only European-like (EUR) individuals [47]. Thus, larger GWAS of opioid sensitivity in multiple genetically inferred ancestry groups are needed to identify genetic predictors of opioid dosing for postoperative pain management.

We used electronic health records (EHR) from the Million Veteran Program (MVP) sample to investigate individual differences in opioid analgesic dosing following knee or hip arthroplasty. These common orthopedic procedures typically cause moderate-to-severe postoperative pain, enabling the study of genetic variation underlying opioid dosing. We aimed to (1) characterize the genetics of inpatient opioid dosing among arthroplasty patients and (2) examine how polygenic scores based on the GWAS are associated with psychiatric, medical, and behavioral phenotypes.

We conducted a GWAS of estimated average daily morphine milligram equivalent (MME) doses prescribed to 27,903 patients who underwent knee or hip arthroplasty. We limited the study to opioids prescribed during the inpatient stay to characterize individual differences in acute post-operative pain management needs. Identifying genetic variation associated with opioid dosing could support more personalized prescribing guidelines and reduce the risk of inadequate pain control and adverse effects, including OUD.

## Methods

All study procedures were approved by both the VA Central Institutional Review Board (IRB) and all local IRBs. Participants provided written informed consent and were not paid to participate.

### Phenotyping

**Figure 1:**
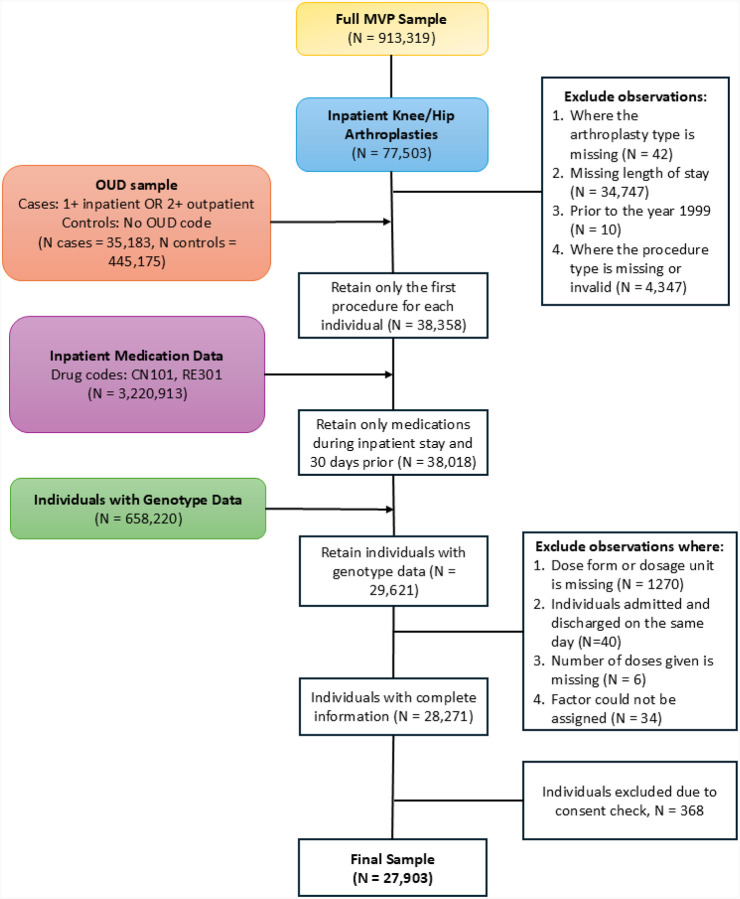
Schematic of the phenotype workflow

We identified patients who underwent knee or hip arthroplasty procedures using International Classification of Diseases (ICD)-9 and ICD-10 diagnoses and Current Procedural Terminology (CPT) codes recorded in the EHR linked to the MVP (see Supplementary Table S1 for a list of diagnosis codes). We excluded arthroplasty procedures performed before 1999 to ensure adequate availability of EHR data. For patients who underwent multiple arthroplasties, we included only the first in the analysis. The length of the hospital stay (in hours) was calculated as the difference between the times of admission and discharge. See Figure 1 for a schematic of the phenotype workflow.

We identified a subset of arthroplasty patients with OUD based on a medical history of at least one inpatient or two outpatient ICD-9 or ICD-10 diagnostic codes for the disorder (Supplementary Table S1). Individuals were considered to have pre-existing OUD if they received the diagnosis prior to or during the hospitalization for arthroplasty.

Data on inpatient opioid medications were extracted from pharmacy records using drug class codes “CN101” and “RE301” from the National Drug Code classification system used by the VA (Supplementary Table S1). We also ascertained whether patients received prescription opioids during the 30 days prior to the arthroplasty admission date for inclusion as a covariate. We excluded prescriptions with missing drug names or drug class information. For each patient, we estimated the average daily morphine milligram equivalent (MME) doses using the following formula:

$$\text{average}\;\text{daily}\;\text{MME}\;\text{doses}=(\left(\text{Number}\;\text{of}\;\text{doses}\;\text{given}\;\times \;\text{MME}\;\text{conversion}\;\text{factor}\;\text{of}\;\text{the}\;\text{drug}\;\text{administered}\right)\;\text{X}\;24)/(\text{length}\;\text{of}\;\text{stay}\;(\text{in}\;\text{hours})$$

We used the number of doses rather than the drug dosage, as the latter value could not always be accurately ascertained from the EHR. We used a publicly available MME conversion table (https://pain.ucsf.edu/opioid-analgesics/calculation-oral-morphine-equivalents-ome) to estimate the MME for each drug type. Because accurate application of the conversion factor required knowledge of both the number of doses and the drug formulation, prescriptions missing either of these were excluded. We summed up the opioid MME doses administered across the entire inpatient stay for each patient and divided this by the duration of the stay in hours to obtain a single average MME doses per hour for each patient, which we multiplied by 24 to get the mean daily MME doses, the phenotype used in GWAS.

### Genotyping and Imputation

Samples were genotyped using a custom Affymetrix Axiom Biobank Array. Quality control (QC) and imputation of genotype data were performed by the MVP working group [27]. QC included the removal of samples for participants whose genetic and phenotypic sex did not match, who had seven or more relatives in MVP (kinship > 0.08), or who demonstrated excessive heterozygosity or a genotype call rate <98.5%. Monomorphic variants, variants with a Hardy-Weinberg equilibrium (HWE) P-value <1 × 10^−6^ or a high missingness (call rate <0.95) were removed. Genotypes were phased using SHAPEIT4 [15] and imputed using Minimac4 software [16]. A combination of the 1000 Genomes Phase 3 panel and the African Genome Resources reference panel was used to impute biallelic SNPs. Genetic ancestry was determined by calculating GIA composition with principal component analysis, as previously outlined in Hunter-Zinck et al. (2020).

### Genome-wide association studies

We performed GWAS stratified by arthroplasty type (i.e., knee or hip) because these procedures differ in surgical approach, recovery time, and patient outcomes [12]. Analyses were also separated by ancestry group (i.e., European-like (EUR), African-like (AFR), and Admixed American-like (AMR)). Linear regression models were implemented using PLINK v.2.0, with age, sex, the first 10 within-ancestry principal components (PCs), and pre-existing OUD diagnosis included as covariates. Variants with an imputation quality (INFO) score <0.8 were excluded. A minor allele frequency (MAF) threshold of 5% was applied to the AFR and AMR GWAS because of their smaller sample sizes, with an MAF of 1% used for the EUR GWAS. To account for relatedness, we randomly removed one individual from each pair of related individuals (kinship >0.08).

Lead SNPs were identified using a two-step linkage disequilibrium (LD) clumping process in the Functional Mapping and Annotation of Genome-Wide Association Studies (FUMA) interface [50]. First, SNPs with P-value ≤5 × 10^−8^ and independent at r^2^ <0.6 were selected as independent significant SNPs, with lead SNPs defined as those independent from each other at r^2^ <0.1, and candidate SNPs defined as those in LD (r^2^ ≥0.6) with lead SNPs and with a P-value ≤0.05. We used the 1000 Genomes reference panel to estimate the r^2^ between SNPs. [4]

### Cross-ancestry and cross-phenotype meta-analyses

Summary statistics from the within-ancestry knee and hip arthroplasty GWAS were meta-analyzed (e.g., EUR hip and EUR knee) using the inverse-variance weighted approach in METAL [51], followed by a cross-ancestry GWAS meta-analysis (i.e., AFR hip/knee meta-analysis with AMR and EUR hip/knee meta-analyses).

### Biological characterization

Findings from each GWAS were characterized biologically using an ensemble of prioritization tools available in FUMA. Lead SNPs were mapped to genes using both positional proximity and functional annotations, including eQTL and chromatin interaction data, to identify connections between genomic regions. [6,33,42]. We used Multi-marker Analysis of GenoMic Annotation (MAGMA) to conduct gene-based enrichment analysis and test for associations between GWAS signals and gene expression data. Following biological characterization, we identified prior associations of the SNPs in the GWAS catalog [9].

### Polygenic scores and phenome-wide association studies

Using PRS-CS software [19], we calculated ancestry-specific polygenic scores (PGS) in the Yale-Penn [28] and Penn Medicine Biobank (PMBB) [49] samples. We did not calculate PGS for AMR individuals because of the small sample sizes of this group in the target cohorts. In PRS-CS, default values were used to estimate shrinkage parameters, except for *phi*, the global shrinkage parameter, which was learned from the datasets. We conducted phenome-wide association studies (PheWAS) using linear and logistic regression models to examine associations between the average daily MME doses PGS and available psychiatric, behavioral, and medical phenotypes, with age, sex, and the first 10 PCs as covariates. We examined the association of PGS with 691 phenotypes (P_Bonferroni_ = 7.23 × 10^−5^) in the Yale-Penn sample, and 1,151 phecodes (P_Bonferroni_ = 4.34 × 10^−5^) for AFR individuals and 1,370 phecodes (P_Bonferroni_ = 3.65 × 10^−5^) for EUR individuals in the PMBB. Fewer phecodes were available for use in AFR individuals because of the requirement that phecodes have ≥100 cases to be included. The Yale-Penn and PMBB samples and genotyping procedures are provided elsewhere [22,28,41].

## Results

### Sample characteristics

The combined sample comprised 27,903 individuals who were predominantly male (92.66%) with a mean (SD) age of 65.95 (9.02). The knee arthroplasty sample comprised 18,540 individuals (mean (SD) age = 66.00 (8.63), 92.20% males), while the hip arthroplasty sample included 9,363 individuals (mean (SD) age = 66.11 (9.74), 93.56% males). The knee arthroplasty sample included 14,088 EUR, 2,846 AFR, and 1,829 AMR individuals. For hip arthroplasty, the corresponding numbers of individuals were 7,012 EUR, 1,606 AFR, and 522 AMR. The overall percentage of individuals with a lifetime diagnosis of OUD was 4.15%, with 3.77% and 4.90% in the knee and hip arthroplasty samples, respectively. Sample characteristics for each genetically inferred ancestry group are presented in Table 1.

### GWAS results

**Figure 2:**
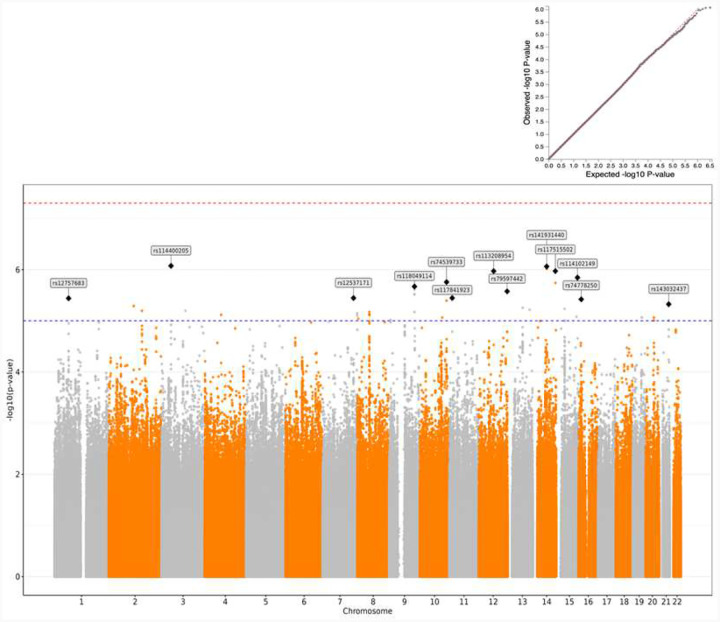
Cross-ancestry and cross-phenotype genome-wide association results for estimated average MME opioid doses in who had a knee or hip arthroplasty. The Manhattan plot displays the −log_10_(P) values of SNP associations across the genome. The red horizontal line indicates the genome-wide significance threshold (P = 5 × 10^−8^), and the blue line marks the suggestive significance threshold (P = 1 × 10−^6^). SNPs exceeding the suggestive threshold are labeled with their rsIDs. The plot in the upper right corner shows the quantile–quantile (QQ) plot of observed versus expected P-values.

In the overall cross-ancestry GWAS meta-analysis, although no loci reached genome-wide significance (GWS; P <5.0 × 10^−8^), 14 loci surpassed the nominal significance threshold (P <5 × 10^−6^). The most significant association was at rs114400205 (P = 8.42 × 10^−7^; see Table 2 and Figure 2).

In the EUR GWAS (Supplementary Table S3, Supplementary Figure 2), no loci were GWS for either phenotype. For the knee arthroplasty GWAS, 11 independent loci were nominally significant, with the lead variant being rs117515502 (P = 2.29 × 10^−7^. For the hip arthroplasty GWAS, 21 loci were nominally significant, with the top SNP being rs115692385 (P = 2.87 × 10^−7^).

Although in the AFR knee arthroplasty GWAS (Supplementary Table S4, Supplementary Figure 3) no variants were GWS, 8 loci were nominally significant (P <5 × 10^−6^), with the lead SNP being rs6727808 (P = 1.63E × 10^−7^). Two loci were GWS in the AFR hip arthroplasty GWAS: one on chromosome 2 (rs116314843, P = 7.59 × 10^−9^) and one on chromosome 3 (rs151228165, P = 2.58 × 10^−9^).

In the AMR knee arthroplasty GWAS, although no loci were GWS, 4 were nominally significant (P < 5 × 10^−6^) (Supplementary Table S5, Supplementary Figure 5). In the hip arthroplasty GWAS, three variants were GWS: rs72773845 (P = 2.97E × 10^−9^), rs6563008 (P=2.49E × 10^−8^) and rs11022449 (P=4.85 × 10^−8^). These were on chromosomes 10, 13, and 11, respectively.

### Biological characterization

In AFR individuals, a SNP that was GWS in the hip arthroplasty GWAS, rs151228165, maps to *GM2AP*. The gene encodes a GM2 activator protein that enables the degradation of GM2 ganglioside, a lipid contained in neuronal membranes [25]. The second GWS SNP, rs116314843, maps to transcript AC125238.3 of the *CD28* gene, which encodes a protein that plays a vital role in T-cell activation, proliferation, survival, and the maintenance of immune homeostasis.

Of the three SNPs that were GWS in the AMR hip arthroplasty GWAS, rs72773845 maps to *SFMBT2*, which encodes a protein involved in chromatin organization and epigenetic regulation [52]. The second SNP, rs6563008, maps to *SCEL*, whose protein product is involved in epidermal differentiation and intercellular adhesion of epithelial cells. The third SNP, rs11022449, maps to *TEAD1*, which encodes a transcription factor known for its role in cell growth and the development of organs, including the heart, skeletal muscle, and nervous system (Gessler et al., 2024; Pelenyi et al., 2024; Song et al., 2024). An examination of nominally significant associations showed several SNPs that map to genes of biological relevance. For example, rs12722489, nominally significant in the AMR knee arthroplasty GWAS, maps to *IL2RA*, which encodes a protein that plays a role in immune tolerance [35]. Using MAGMA gene property analysis, we examined the relationship between tissue-specific gene expression and gene-level association signals from the cross-ancestry meta-GWAS. The only significant association in the datasets tested was in brain tissue from the early infancy developmental stage in the BrainSpan data (P = 1.09 × 10^−03^; Supplementary Table S7, Supplementary Figure 7).

### PheWAS in the PMBB and Yale-Penn Sample

PheWAS conducted using summary statistics from the EUR and AFR GWAS to calculate PGs in the target samples yielded several associations, though none survived Bonferroni correction. The EUR knee arthroplasty PGS was nominally associated with 79 traits in the PMBB. Among these, higher PGS were nominally associated with staphylococcal infections (beta = 0.235, SE = 0.061, *p* = 1.02 × 10^−4^), major depressive disorder (beta = 0.092, SE = 0.025, *p* = 2.33 × 10^−4^) and anxiety disorders (beta = 0.061, SE = 0.019, *p* = 1.41 × 10^−3^). For the EUR hip arthroplasty PGS, 65 associations were nominally significant in the PMBB. In AFR PMBB participants, knee and hip arthroplasty PGS were nominally associated with 41 and 63 traits, respectively (Supplementary Table S8, Supplementary Figure 8).

In the Yale-Penn sample, 14 phenotypes were nominally associated with knee arthroplasty PGS in EUR individuals, eight of which were substance related. Other psychiatric traits were also nominally associated with the knee arthroplasty PGS in EUR individuals, including lifetime trauma assessment (beta = −0.079, SE = 0.029, *p* = 6.90 × 10^−3^) and trouble functioning due to depression (beta = 0.061, SE = 0.029, *p* = 3.77 × 10^−2^). There were 49 nominal associations with the hip arthroplasty PGS in EUR Yale-Penn participants, 36 of which were substance related. In the AFR Yale-Penn participants, there were 42 nominal associations for the knee arthroplasty PGS and 28 for the hip arthroplasty PGS (Supplementary Table S9, Supplementary Figure 9).

## Discussion

This study is the first genome-wide investigation of variation in inpatient opioid dosing following knee or hip arthroplasty. Utilizing data from the MVP cohort, a large, diverse biobank comprising >600,000 genotyped individuals with EHR data, we identified 27,903 individuals who underwent one of these surgeries. The findings enhance our understanding of the genetic underpinnings of individuals’ opioid analgesic dosing needs and advance the field beyond traditional candidate gene approaches [7,13,17,31,38] and small-sample GWAS (Aoki et al., 2015; Cook-Sather et al., 2014; Nishizawa et al., 2014) that have been used to study these effects.

We identified 5 GWS loci, three in AMR individuals and two in AFR individuals. Two of the three loci in the AMR hip arthroplasty GWAS mapped to genes previously implicated in a GWAS of opioid dependence: *SFMBT2* and *RPL17P35* [10,18]. Although there were no GWS loci identified in either the cross-ancestry meta-analysis or the EUR GWAS, these analyses yielded many nominally significant loci. Of the genes mapped to nominally significant loci across all the GWAS that we conducted, 19 were previously associated with traits related to pain intensity, response to opioids, osteoarthritis, or the revision of knee or hip arthroplasties. For example, four genes identified in our study, *GRIA1*, *NRXN1*, *ZNF423*, and *ROBO2*, were also identified in a GWAS of pain intensity in the MVP sample [48]. These genes’ associations with pain intensity may contribute to their link to opioid dosing. Notably, tissue enrichment analysis showed significant enrichment in developmental tissue from early infancy, suggesting that genetic variation related to opioid dosing may also be involved in mechanisms that support the structural and functional development of the brain, as has previously been reported [21]. Specifically, the anterior cingulate cortex (ACC) area BA24 was the most enriched tissue type, consistent with prior studies highlighting the role of this brain region in pain transmission and processing [2,30].

As with other complex traits, post-operative opioid dosing is likely highly polygenic, with small effects distributed across the genome. Detecting such effects will require larger samples than those used here. Another limitation of the present study is the phenotype used—an estimate of MME dosage based on the number of doses of different opioids administered during a discrete period, but not the dosage of each. Because this approach, necessitated by the distributed nature of pharmacy records in the VA EHR, does not fully capture the variation in opioid dosing, efforts are needed to capture more detailed opioid dosing information. Third, the impact of other factors, such as recent changes in opioid prescribing patterns, provider preferences, patient perception of pain, and social biases, may also influence which opioids are prescribed to patients and their dosage following surgery. Thus, studies that consider these factors’ effects on opioid dosing are also needed [26,29,32,34]. A potential limitation of our findings is collider bias. Because all individuals in our sample underwent a knee or hip arthroplasty, the genetic and phenotypic associations may partially reflect factors related to the need for an arthroplasty in addition to opioid dosing requirements specifically. We also saw key differences in the phenotypes associated with the arthroplasty PGS between the PMBB and Yale-Penn sample. These differences are likely because the PMBB is a biobank linked to an EHR with a broad swath of diagnostic and procedural data from both inpatients and outpatients, while the Yale-Penn sample was ascertained and deeply phenotyped in the context of genetic studies of substance use disorders.

Despite these limitations, our study has several key strengths. It is the first to use GWAS to identify genetic predictors of opioid dosing in the period following arthroplasty, a surgical procedure associated with high levels of pain. To do this, we leveraged a large and genetically diverse biobank—MVP—with the goal of characterizing opioid prescribing patterns in an inpatient setting. Whereas MVP patients were prescribed a wide variety of opioids comprising multiple drug formulations, we maximized the available information using an MME conversion factor to yield a consistent measure across individuals. Following GWAS, we performed biological annotation to gain insights into the functional importance of identified loci and evaluated the predictive performance of our findings by generating PGS in two distinct, independent samples. These results provide a foundation for future work aimed at advancing the precision treatment of post-operative pain and highlight the importance of large, diverse datasets for uncovering genetic contributors to prescription opioid dosing.

## Supplementary Material

This is a list of supplementary files associated with this preprint. Click to download.

- Table1.xlsx
- Table2.xlsx
- SupplementalInformation.docx
- SupplementaryTables.xlsx
- SupplementaryFigures.docx

## Data Availability

The cross-ancestry and within-ancestry GWAS and meta-analysis summary-level association data will be available in the database of Genotypes and Phenotypes (dbGaP) (https://www.ncbi.nlm.nih.gov/gap/) under accession phs001672 “Veterans Administration (VA) MVP Summary Results from Omics Studies.” Registration and approval are needed following dbGaP’s data access process.
